# Improving lives by linking data: Views from groups with complex needs. Commissioned by the cross-government Better Outcomes through Linked Data programme and the Centre for Data Ethics and Innovation, and delivered by the research agency Britain Thinks

**DOI:** 10.23889/ijpds.v8i2.2320

**Published:** 2023-09-14

**Authors:** Dean Fell, Loulwa Al Rasheed-Wright

**Affiliations:** 1 Ministry of Justice, London, United Kingdom; 2 Thinks Insight, London, United Kingdom

## Objectives

- Improve and refine BOLD's delivery of its four pilot projects via feedback on acceptable uses and forms of analysis of shared data.
- Inform how BOLD communicates its aims and progress for maximum clarity and transparency.
- Understand the impact of BOLD's data management on the level of trust in the program.

## Methods

Initial workshops were conducted with expert intermediary organisations, such as charities, who have a deep understanding of each of the audiences’ attitudes and needs. This was to explain the purpose of the project to the intermediary organisations and to gain their feedback on the approach and materials.

Fieldwork was undertaken with 82 participants from across the four vulnerability audiences, using a combination of methods to ensure full participation from a range of individuals with differing levels of need.

Participants were presented with information about BOLD's purpose, objectives, and three example scenarios related to their respective pilot to prompt useful discussions.

## Results

- Participants in the BOLD program pilots generally support data sharing to improve public services.
- They view the program positively, have similar experiences across pilot areas, and reflect on its relevance to a range of audiences.
- However, some lack experience with issues addressed by BOLD. Concerns exist regarding negative impacts and scepticism towards government.
- BOLD use cases should prioritise four principles: relevance, impact, clarity, and non-stigmatisation.
- Intermediary organisations are crucial for engaging with pilot audiences.

## Conclusion

BOLD is a three-year cross government pilot project that links social policy datasets to better support individuals with complex needs. It aims to improve outcomes by providing new evidence and insight into how services delivered in one part of government affect outcomes in another. This research was commissioned to inform how to take the BOLD programme forward, by engaging and consulting with relevant audiences in an ethical and transparent way.

The published findings are informing the delivery of BOLD and improving and enhancing our understanding of the impact of BOLD's data management on trust.

